# A pictorial presentation and the clinical use of the modified trauma axial (MTA) shoulder x‐ray view

**DOI:** 10.1002/jmrs.670

**Published:** 2023-03-20

**Authors:** Yat Hang Tam, Dania Abu Awwad

**Affiliations:** ^1^ Concord Repatriation General Hospital Radiology Department Sydney NSW Australia; ^2^ Discipline of Medical Imaging Science, Faculty of Medicine and Health The University of Sydney Camperdown NSW Australia

**Keywords:** axillary radiographs, Bankart lesions, Hill‐sachs lesions, post‐reduction x‐rays, radiography, shoulder dislocation

## Abstract

Anteroposterior (AP) and lateral shoulder projections are routinely performed as part of a post‐reduction shoulder x‐ray series in the emergency department (ED). Research has shown that these projections alone are insufficient to demonstrate post‐dislocation injuries, particularly Hill‐Sachs and Bankart lesions. These concomitant pathologies are best demonstrated on axial shoulder projections but are difficult to obtain in trauma patients with limited range of motion. The diagnostic quality and the pathology demonstrated by different projections is crucial so that doctors and other ED staff can triage patients appropriately, radiologists can report on the presence or absence of post‐dislocation shoulder injuries, and the orthopaedic team can plan for follow‐up or treatment. Different modified axial views were reported to improve the post‐dislocation pathology sensitivity in the shoulder series. However, all of these shoulder axial views require patient movement. The modified trauma axial (MTA) is an alternative projection that is suitable for trauma patients that does not depend on patient movement. This paper presents several cases where the MTA shoulder projection had clinical importance when used as part of the post‐reduction shoulder series in the ED or radiology department.

## Graphical abstract

The modified trauma axial (MTA) is an axial shoulder projection used to demonstrate shoulder dislocation related injuries to the humeral head and glenoid. It does not require patient movement and is easily implemented in an acute trauma situation. This pictorial essay presents several case studies where the MTA projection has been an important addition in the post‐reduction shoulder series.

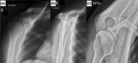

## Introduction

The glenohumeral joint is a commonly dislocated joint, and recurrent dislocations are a frequent problem.^1^ Soft tissue and bony injuries may occur during a patient's initial dislocation, leading to increased shoulder instability.^2^ Anterior dislocations make up 97% of all shoulder dislocations, where the humeral head moves anteriorly and medially in relation to the glenoid fossa.^1^ The posterolateral aspect of the humeral head impacts the anterior glenoid rim, which can also lead to compression fractures posteriorly called a Hill‐Sachs lesion.^3^ Additionally, the forced movement of the humeral head anteriorly can cause damage to the labrum and glenoid rim, causing a Bankart lesion and an associated bony Bankart.^3^ A Bankart lesion occurs when the anterior aspect of the glenoid labrum is torn or avulsed and may also avulse a portion of the glenoid bone which is referred to as a bony Bankart.^4^

Radiographs before reduction are beneficial, particularly if it is the patient's first dislocation, as fractures are more commonly seen in the first dislocation than in recurrent incidences.^3^ Hill‐Sachs lesions have been reported in 40%–90% of initial cases of anterior shoulder dislocations.^1^ They are also typically found with other abnormalities, such as Bankart and bony Bankart lesions.^1^ Post‐reduction radiographs are beneficial to confirm shoulder relocation and to diagnose any missed associated fractures.^3^ It is common to perform an anteroposterior (AP), lateral scapula, and axillary shoulder projection.^3^ A third of fractures associated with shoulder dislocations can only be seen on post‐reduction radiographs, particularly Hill‐Sachs lesions.^3^ Axial shoulder projections are deemed most sensitive to detect bony abnormalities but require rotation or abduction of the arm.^2^, ^5^ Thus, these axial views are challenging to obtain in trauma situations, as patients tend to have limited range of movement, are experiencing considerable pain, or are sedated.^2^, ^3^, ^6^

**Figure 1:**
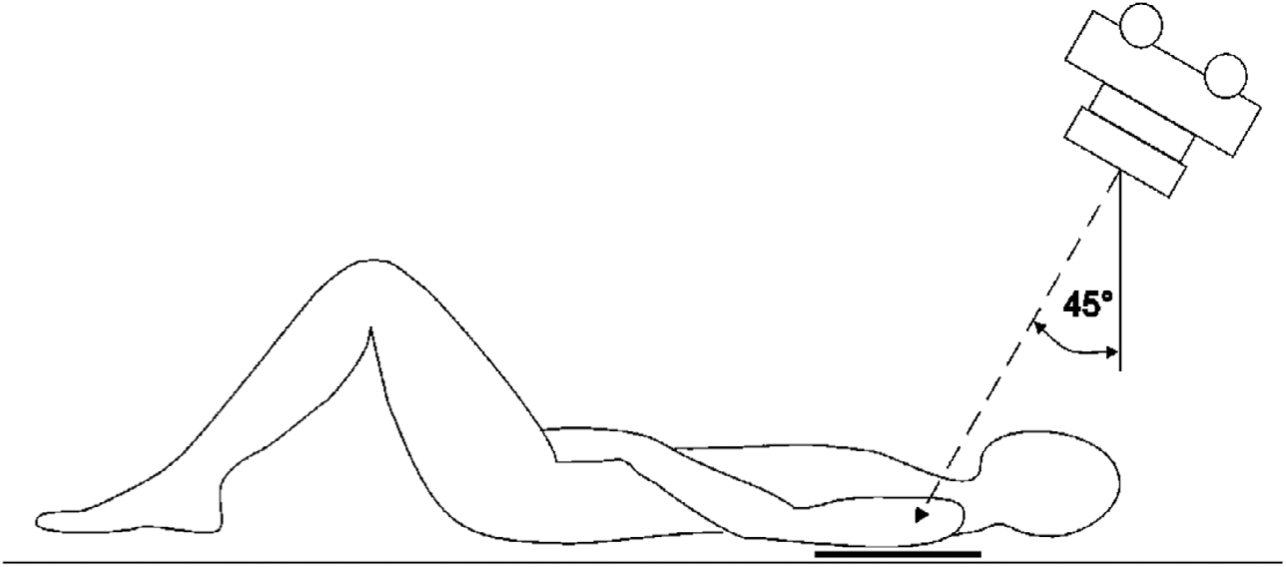
Supine position of the Modified Trauma Axial shoulder projection (Neep & Aziz,^5^ reproduced with permission from Elsevier).

A study conducted by Neep and Aziz found that using the AP and modified trauma axial (MTA) projection detected a significantly higher number of post‐dislocation pathologies than the shoulder AP and lateral scapula projections, particularly for greater tuberosity avulsions, Hill‐Sachs lesions, and glenoid rim fractures.^5^ The MTA requires the patient to be either erect AP or supine with no body rotation. The x‐ray tube is angled 45° caudally and centred on the glenohumeral joint (see Fig. 1).^5^ Radiographically, the MTA demonstrates an elongated view of the coracoid process, glenoid, and acromion and is also useful for visualising the articulation of the humeral head and glenoid fossa.^5^ The demonstration of these structures is essential in the context of shoulder dislocations because there is a statistically significant relationship between the formation of Hill‐Sachs lesions and recurrent dislocations.^1^, ^3^ The size of the Hill‐Sachs lesion and the presence of glenoid rim fractures are important considerations for surgical intervention.^1^, ^7^ There remains a debate over whether radiographs are required for shoulder reductions,^8^, ^9^ but the following four case studies support the need for performing MTA views as part of the post‐reduction shoulder series. The perspectives and interpretations of an emergency department (ED) doctor, radiologist, and orthopaedic doctor for each case study were also included. These specialists viewed the case studies and provided commentary for each radiograph. It is important to note that the ED team will typically review the radiographs and facilitate appropriate care before they receive the radiology report. Their interpretations have been synthesised in the case study descriptions below. Ethical exemption was granted by the Sydney Local Health District Human Research Ethics Committee for the use of deidentified radiographs.

## Case studies

### Case study 1

**Figure 2:**
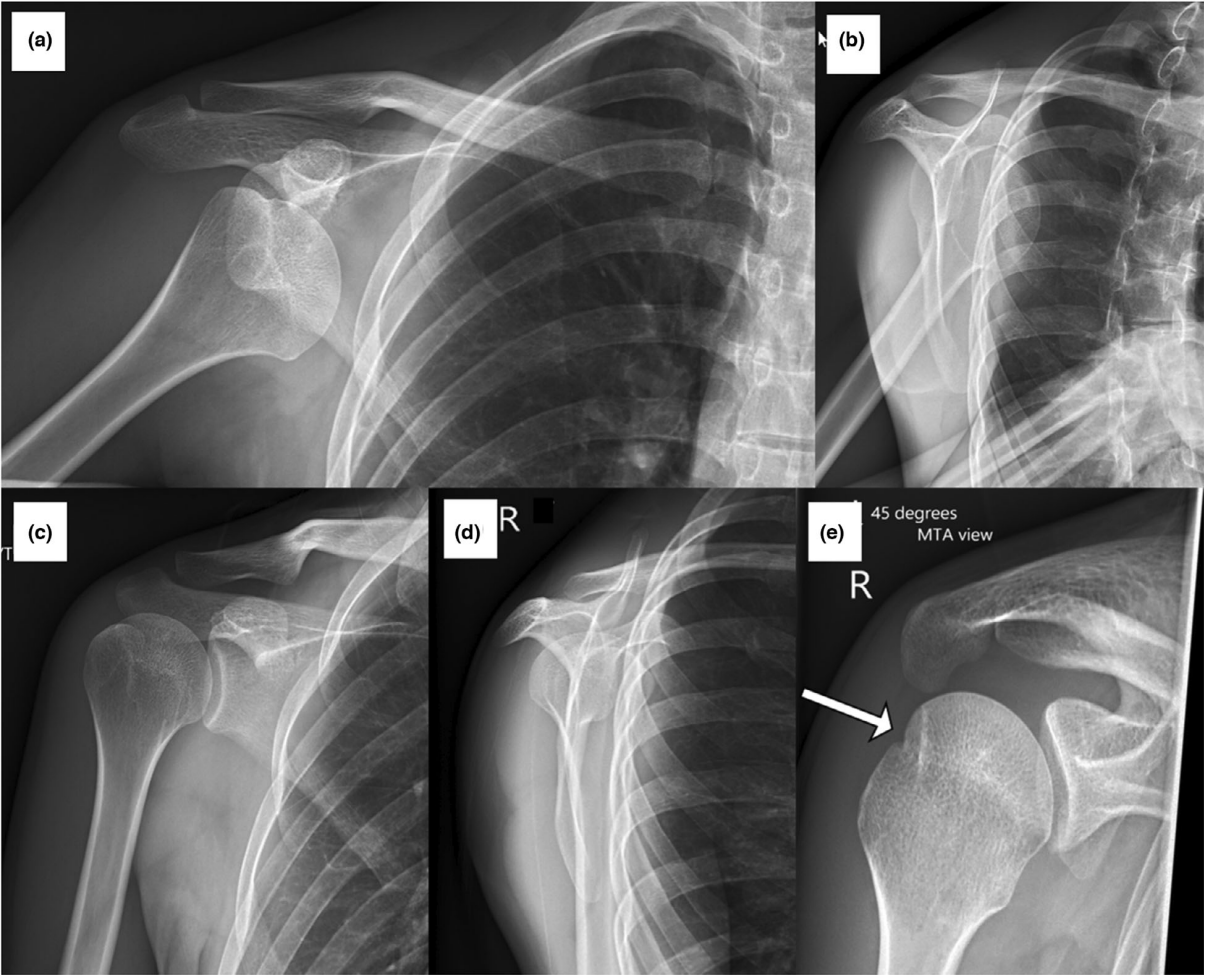
The patient presented for query dislocation. Radiographs were taken pre (A,B) and post (C–E) shoulder reduction. AP (A) and lateral scapula (B) shoulder projections both show anterior dislocation. The post‐reduction AP (C) and lateral scapula shoulder (D) radiographs show no evidence of abnormality. Image E is the MTA view, and a Hill‐Sachs lesion is demonstrated (arrow). MTA, Modified trauma axial.

The patient presented to ED for a query dislocation and pre‐ and post‐reduction radiographs were taken (Fig. 2). The initial radiographs confirmed the presence of an anterior dislocation. The post‐reduction radiographs confirmed that the humeral head had been relocated correctly. When asked about individual radiographs, an ED doctor, radiologist, and orthopaedic doctor stated that the AP and lateral scapula shoulder radiographs alone did not show any obvious bony injuries, but the MTA confirmed the presence of a Hill‐Sachs lesion. This confirmation allowed the patient to be recommended for orthopaedic review.

### Case study 2

**Figure 3:**
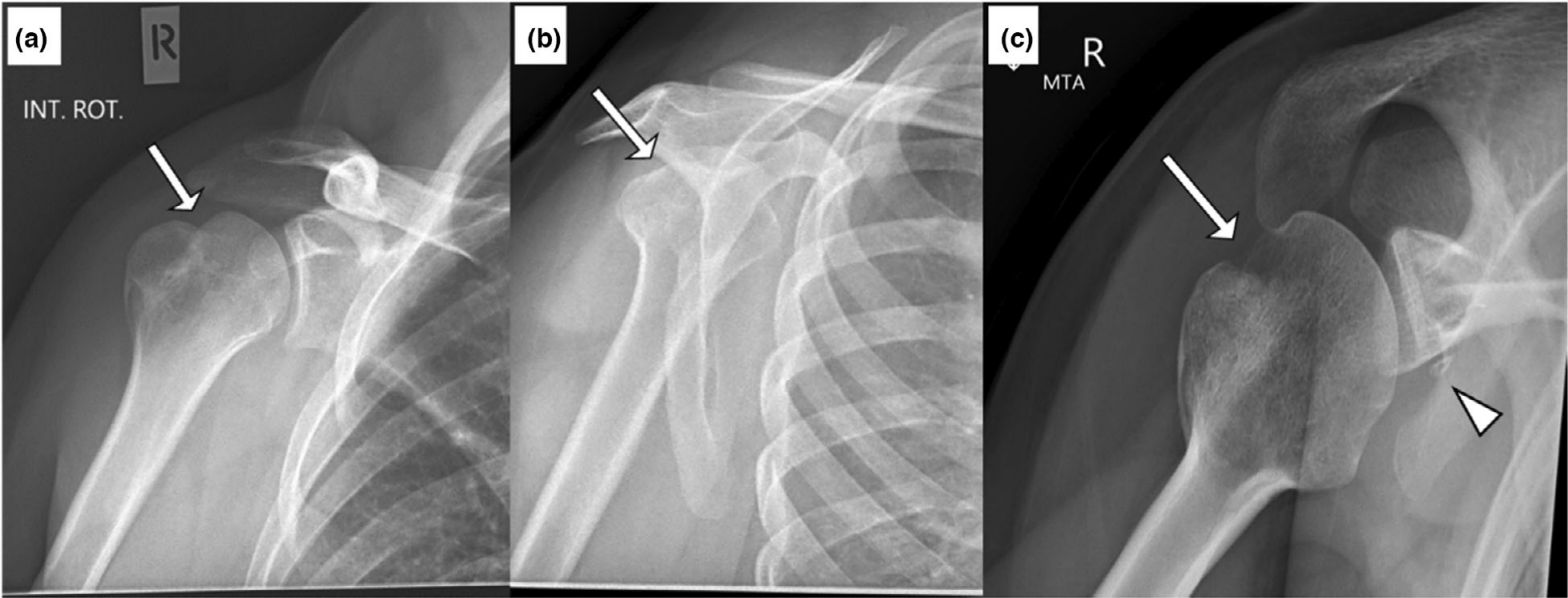
The patient with a recent shoulder dislocation presented to the radiology department for progress x‐rays and a known greater tuberosity fracture. The Glenoid view (A) shows a Hill‐Sachs lesion (arrow). The lateral scapula shoulder view (B) shows a flattened appearance of the posterior humeral head suggestive of a Hill‐Sachs lesion. The MTA (C) demonstrates the Hill‐Sachs lesion and a small bony Bankart (arrowhead). MTA, Modified trauma axial.

The patient presented to the radiology department for follow‐up imaging with a known history of a shoulder dislocation and greater tuberosity fracture (Fig. 3). On previous imaging, this patient did not have any axial or MTA radiographs taken. On review of individual radiographs, an ED doctor, radiologist, and orthopaedic doctor confirmed that the Hill‐Sachs lesion and the healing greater tuberosity fracture can be seen on all projections. However, the MTA projection showed an additional Bankart lesion that was not seen on the other projections.

### Case study 3

**Figure 4:**
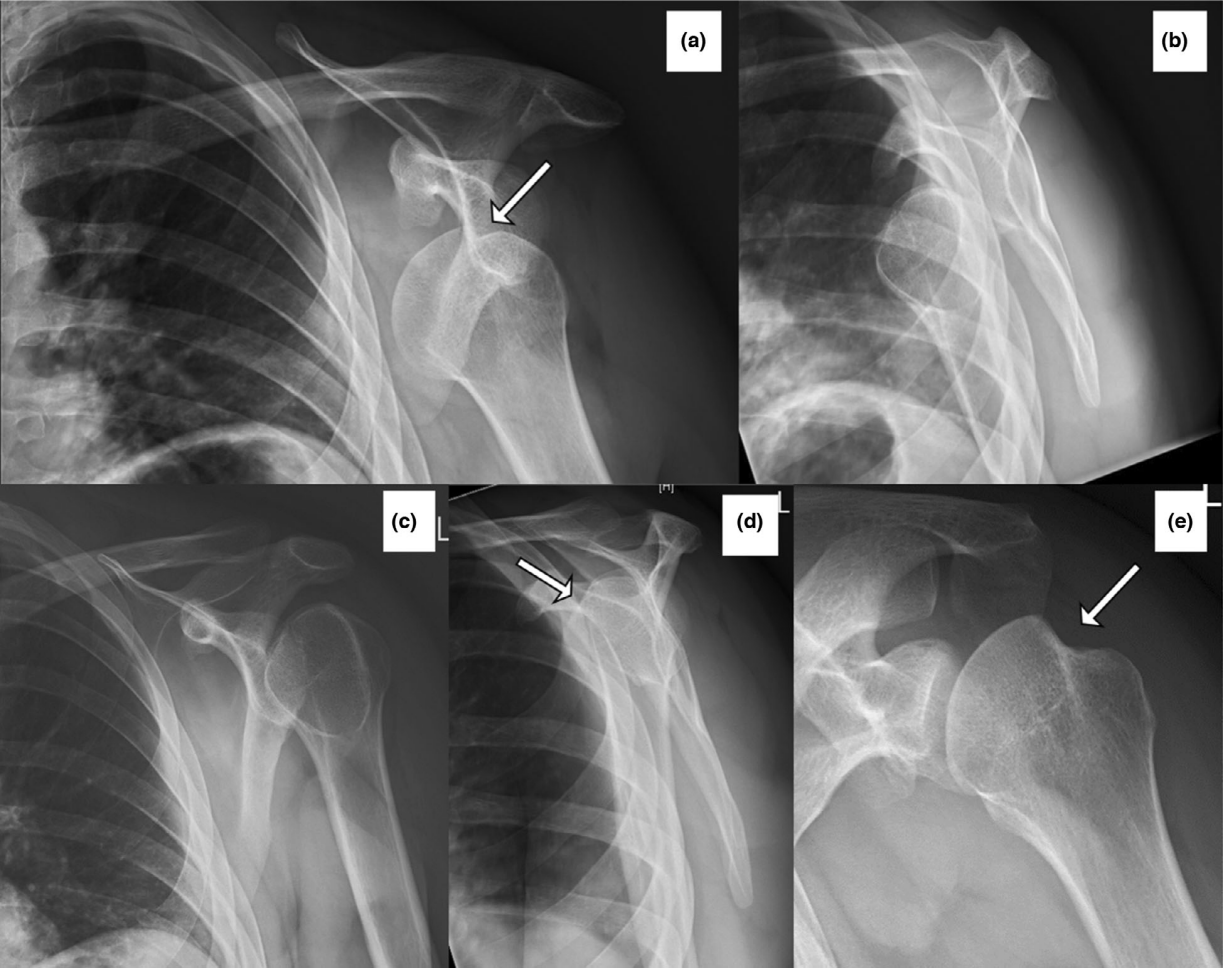
The patient presented with a dislocation to rule out fractures. Radiographs were taken pre (A‐B) and post (C‐E) shoulder reduction. AP shoulder (A) and lateral scapula shoulder (B) show anterior dislocation and slight indentation of the superior humeral head suggestive of a Hill‐Sachs defect. The post‐reduction AP (C), lateral scapula (D), and MTA (E) view show indentation and sclerosis in the superior humeral head. The Hill‐Sachs lesion (arrow) is best demonstrated on the MTA view (E). MTA, Modified trauma axial.

The patient presented to ED with a dislocated shoulder and radiographs were requested pre‐ and post‐reduction (Fig. 4). The Hill‐Sachs lesion can be seen in both pre‐ and post‐reduction radiographs. For the ED doctor, the patient would be referred for orthopaedic review with or without the MTA projection. However, the radiologist stated that the MTA projection demonstrated the Hill‐Sachs lesion much more evidently and with a greater involvement of the humeral head. The orthopaedic doctor echoed the radiologist's comment, adding that the MTA evidently showed the need for further imaging and surgical intervention.

### Case study 4

**Figure 5:**
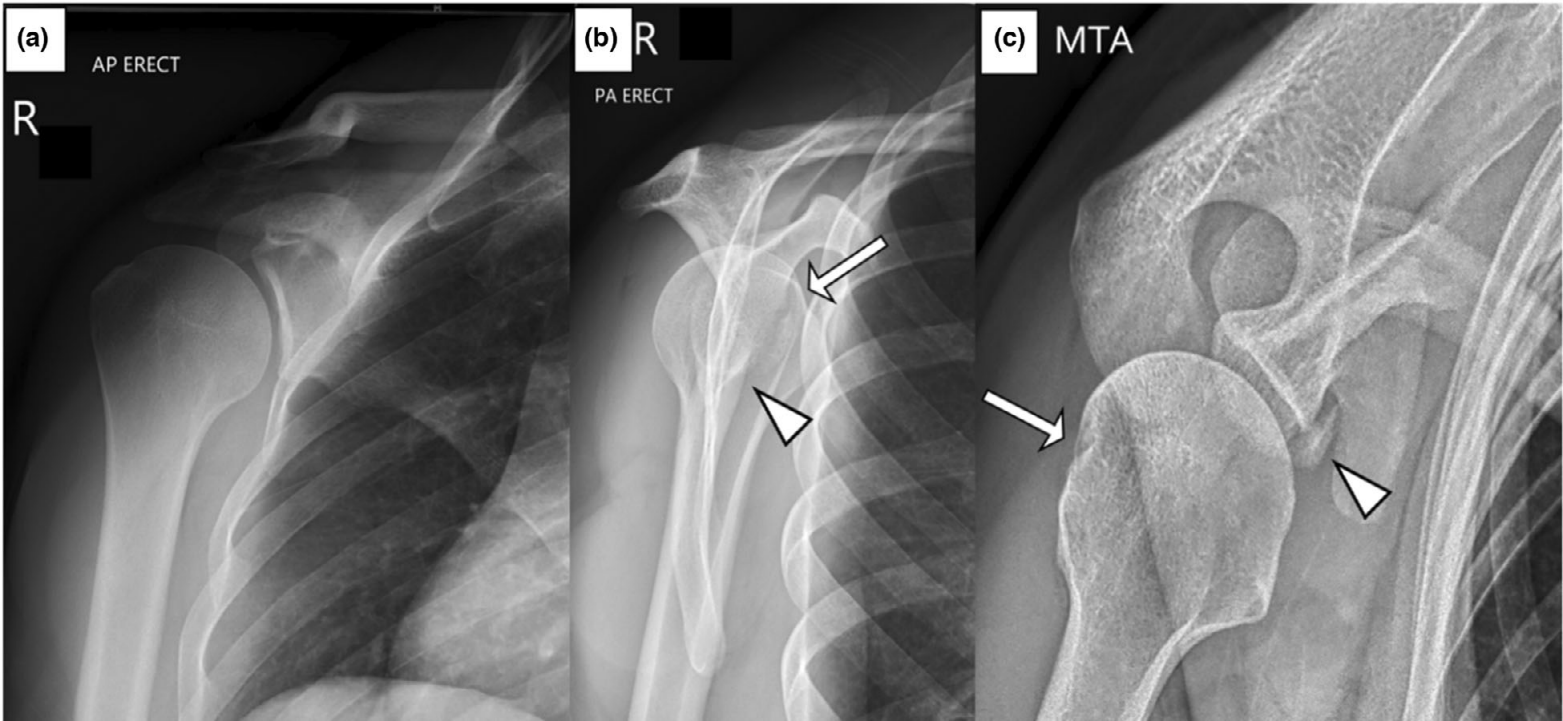
The patient presented to emergency department after a shoulder reduction attempt in the ambulance and no radiographs were taken prior. Glenoid view (A) has no abnormality detected. The lateral scapula shoulder projection (B) has a subtle radiolucency line in the anterior glenoid rim (arrowhead), suggesting a bony Bankart. The MTA view (C) confirms the bony Bankart (arrowhead) and the depression of the posterolateral aspect of the humeral head is the Hill‐Sachs lesion (arrow). MTA, Modified trauma axial.

The patient was taken to ED with a shoulder dislocation that had been relocated in the ambulance (Fig. 5). Hence, no pre‐reduction radiographs were taken. The radiographs confirmed that the glenohumeral joint was enlocated. From the AP glenoid and lateral scapula shoulder projections, the ED doctor stated that there were no obvious bony injuries. Without the MTA projection, the patient would not have been referred for orthopaedic review. The radiologist confirmed that the MTA projection best showed the bony Bankart lesion and Hill‐Sachs lesion, but on review, the radiolucent line on the lateral corresponds to the Bankart lesion (image B, white arrow).

## Discussion

### Hill‐Sachs and Bankart lesions

Most radiology departments routinely perform pre‐ and post‐reduction shoulder radiographs for patients with shoulder dislocations consisting of AP and lateral scapula shoulder projections.^8^ At the study site where the presented case studies were performed, the protocol was either a standard AP or glenoid AP along with the lateral scapula shoulder and MTA projection. The importance of post‐reduction shoulder radiographs in ED has been under debate, along with which fractures are clinically important.^8^, ^9^, ^10^ Gottlieb et al. reported that the post‐reduction radiographs help identify new fractures, but none of the fractures identified are clinically important and change ED management.^8^ These new fractures mostly consisted of Hill‐Sachs and Bankart lesions.^8^ Another study has identified traumatic pathologies in 40% of shoulder radiographs with acute trauma, most of which were Hill‐Sachs lesions.^5^ Despite there being a statistically significant relationship between Hill‐Sachs lesions and recurrent dislocations,^1^, ^3^ Gottlieb et al. argued that Hill‐Sachs and Bankart lesions do not require immediate attention by the orthopaedic team.^8^ Hence, these fractures were deemed clinically insignificant in the ED setting because patients can be seen on follow‐up. Studies have recommended having follow‐up appointments in orthopaedic clinics with post‐reduction shoulder radiographs performed in outpatient clinics instead of in ED to reduce the workflow and burden.^8^, ^9^ This arrangement means that the responsibility of detecting post‐shoulder dislocation pathologies is on outpatient clinics instead of ED. However, for ED settings that does not have this arrangement, the detection of humeral, and glenoid fractures is still important, including Hill‐Sachs and Bankart lesions. This is because ED doctors and staff rely on the findings of the pre‐ and post‐reduction radiographs to facilitate appropriate care and follow‐up with orthopaedic doctors.^10^ In case study 1, the patient had post‐reduction imaging while still in ED. The AP and lateral scapula shoulder projections alone did not warrant further follow‐up, but the presence of the Hill‐Sachs lesion on the MTA projection allowed the patient to be sent for orthopaedic review. On the other hand, case study 2 was an example of a patient presenting for follow‐up imaging as an outpatient without having prior axial shoulder radiographs taken. An MTA projection was taken because of the patient's recent shoulder dislocation, and an additional bony Bankart was seen. In both cases, the additional axial shoulder projection provided more information, whether it was performed immediately in ED or on follow‐up imaging.

### Shoulder radiographic views post‐reduction

Riebe et al. reported that as many as four views (AP projections with internal and external rotation, lateral, and axial) are required to improve the sensitivity of Hill‐Sachs lesions in post‐reduction radiographs.^10^ An AP projection with internal rotation is the most sensitive to demonstrate Hill‐Sachs lesions, while the lateral scapula shoulder projection is the least.^10^ Typically, patients present in a sling with their arm internally rotated, yet, the radiologists have commented that the MTA projection in the case studies has best demonstrated the Hill‐Sachs lesions. Neep and Aziz have also reported that the AP and MTA projections could detect significantly more pathologies than the AP and lateral scapula shoulder views combined.^5^ The authors proposed to replace the lateral scapula shoulder view with the MTA for the post‐reduction radiograph. The case studies in this paper support this proposal. The MTA view is the only view that detected the Hill‐Sachs lesion in case study 1 (Fig. 2), a Bankart lesion in case study 2 (Fig. 3), and a Hill‐Sachs lesion of significant size in case study 3 (Fig. 4). It is important to note that a radiologist acknowledged that no Hill‐Sachs lesion would have been reported without the MTA projection for case study 1 (Fig. 2) and the patient would not have been referred for orthopaedic review by the ED doctors. The same applied to case study 4 (Fig. 5), where the ED doctor would not have sent the patient for orthopaedic review because there were no obvious bony injuries seen on the other projections. The Bankart lesion was evident in the MTA view, subtle in the lateral projection, and superimposed by the ribs in the AP glenoid projection. The study conducted by Neep and Aziz replaced the lateral scapula shoulder projection with the MTA projection and found 10 times more Hill‐Sachs and four times more Bankart lesions.^5^ Different doctors of varying experience will be assessing these images, and Riebe et al. have reported that the accuracy of detecting a Hill‐Sachs lesion is dependent on the experience of the reporting doctors.^10^ Across these case studies, the Hill‐Sachs and bony Bankart lesions were challenging to appreciate on the traditional AP and lateral scapula radiographs. However, the MTA projection has given certainty to the presence of Hill‐Sachs and Bankart lesions in these case studies, and this further highlights the clinical utility of the MTA projections. The lateral scapula shoulder projection presented in the four case studies provided no additional diagnostic value when considering the inclusion of the MTA view. This result is supported by the findings of Neep and Aziz, who reported a statistically significant improvement in the number of shoulder injuries identified on radiographs when the lateral scapula projection was replaced by the MTA.^5^ Another study compared the impact of including one additional axial projection to the shoulder series.^11^ The projection was similar to the MTA view, but the patient is rotated relative to the image receptor. The study found a statistically significant increase in the number of abnormalities identified with the additional axial projection. Specifically, there was an increase in the number of fractures identified, most of which were Hill‐Sachs or fractures to the glenoid.^11^

An accurate diagnosis made from post‐reduction radiographs is crucial for patient management. The addition of the axial projection can impact patient management and reduce the dependence on further imaging to detect shoulder injuries.^11^ The size and classification of the Hill‐Sachs and Bankart lesions impact the treatment plan.^1^, ^7^ Hill‐Sachs lesions involving more than 40% of the humeral head require surgery.^7^ Anything less than that may require surgery depending on the shoulder's stability but can be treated conservatively if it is less than 20%.^7^ The MTA view confirmed the presence of Hill‐Sachs lesions in all four case studies and Bankart lesions in case studies 2 and 4 (Figs. 3 and 5). In addition, the MTA view demonstrated a Hill‐Sachs lesion that potentially has more than 20% involvement in the humeral head case study 3 (Fig. 4), which is a significant clinical indicator for surgical intervention. Looking at the AP or glenoid projections alone, the Hill‐Sachs lesion appearance is very similar between case studies 1, 3, and 4 (Figs. 2, 4, and 5). However, the involvement of the humeral head is significantly different for case study 3 (Fig. 4) than for cases 1 and 4 (Figs. 2 and 5) in the MTA view. Also, once the Bankart lesion has been identified on radiographs, a computed tomography (CT) scan can then be used to quantify glenoid bone loss to aid the treatment plan.^2^ All these observations from the MTA view benefits the orthopaedic doctor's awareness of the patient's condition, resulting in a better treatment plan. However, the initial radiographs assist in determining which patients require orthopaedic review by the ED team to begin with.

Some scholars have reported using the modified axillary view^12^ and the Velpeau view^6^ in acute shoulder trauma because they do not require any patient arm movement while improving the sensitivity of the post‐reduction series. However, these views require patients to stand and lean forward^12^ and backwards^6^ enough to achieve a superior to inferior view of the shoulder, which might not be possible when the patient is unable to move or is sedated. The MTA view does not require any patient rotation or tilt, which is feasible for all patient conditions, especially in ED. For post‐reduction or follow‐up imaging in outpatient clinics prior to their orthopaedic appointment, the West Point^13^ and Stryker notch^14^ projections were reported to best demonstrate Bankart and Hill‐Sachs lesions, respectively.^1^, ^7^ If the patient has regained better mobility, these projections would be attempted over the modified axillary shoulder projections. However, both projections require the shoulder to be abducted either laterally or anteriorly by 90° or more; hence, they are not appropriate for patients following shoulder relocation. Further research to compare the sensitivity of post‐dislocation pathology in the MTA projection to these two views might be beneficial. If the sensitivity of the MTA view is compatible with the West Point and Stryker notch views, it seems an appropriate replacement, particularly for patients with a limited range of movement. The MTA view is easy to perform and has been proven sensitive to several shoulder dislocation pathologies, making it a reliable alternative or replacement for post‐reduction projections.^5^

### Limitations

One limitation of the MTA view that warrants consideration is the use of a 45° caudal angle of the x‐ray tube. Therefore, the anatomy of the MTA view is elongated. Thus, the image is not suitable for measurement. However, this should not stop one from using the MTA view because a CT scan is the imaging method of choice for the quantification of Hill‐Sachs and Bankart lesions after initial radiographs.^2^

Lastly, further research on the clinical impact of the MTA view with a larger cohort is needed. An MTA view in‐service training for ED staff, radiologists, orthopaedic doctors, and radiographers may be required to understand its application and use within the post‐reduction shoulder series.

## Conclusion

The MTA plays an important role in demonstrating shoulder dislocation pathologies in the case studies presented in this pictorial essay. The MTA view alone detected a Hill‐Sachs lesion and Bankart lesion in case studies 1 (Fig. 2) and 2 (Fig. 3), respectively. The MTA view also assisted the radiologist in better appreciating the size of the Hill‐Sachs lesion (case study 3, Fig. 4) and the Bankart lesion (case study 4, Fig. 5). The ability of the MTA view to clarify the size and presence of the Hill‐Sachs and Bankart lesions ensures patients are referred for appropriate follow‐up care and may improve the accuracy of radiologists' reports.

## Conflict of interest

No funds, grants, or other support were received. The authors declare no conflict of interest.
